# Surgical ablation of long-standing persistent atrial fibrillation: 1-year outcomes from the CArdioSurgEry Atrial Fibrillation (CASE-AF) registry

**DOI:** 10.1093/icvts/ivad203

**Published:** 2023-12-13

**Authors:** Herko Grubitzsch, Etem Caliskan, Taoufik Ouarrak, Jochen Senges, Nicolas Doll, Michael Knaut, Thorsten Lewalter, Walter Eichinger, Bernd Niemann, Ivar Friedrich, Torsten Hanke, Volkmar Falk, CASE-AF Investigators, Thorsten Hanke, Thorsten Lewalter, Jochen Senges, Herko Grubitzsch, Etem Caliskan, Taoufik Ouarrak, Michael Knaut, Walter Eichinger, Bernd Niemann, Ivar Friedrich, Volkmar Falk, Mahmoud Wehbe, Marc Albert, Maximilian Vondran, Tamer Ghazy, Henning Warnecke, Mirko Doss, Andreas Liebold, Edgar Eszlari, Ardawan Rastan, Ivana Mitrovic, Adi Cvorak, Theodor Fischlein, Falk-Udo Sack, Gerd Hindricks, Nicolas Doll

**Affiliations:** Department of Cardiothoracic and Vascular Surgery, Deutsches Herzzentrum der Charité (DHZC), Berlin, Germany; Charité—Universitätsmedizin Berlin, corporate member of Freie Universität Berlin, Humboldt-Universität zu Berlin, and Berlin Institute of Health, Berlin, Germany; Department of Cardiothoracic and Vascular Surgery, Deutsches Herzzentrum der Charité (DHZC), Berlin, Germany; Charité—Universitätsmedizin Berlin, corporate member of Freie Universität Berlin, Humboldt-Universität zu Berlin, and Berlin Institute of Health, Berlin, Germany; Institut für Herzinfarktforschung, Ludwigshafen, Germany; Institut für Herzinfarktforschung, Ludwigshafen, Germany; Department of Cardiac Surgery, Schüchtermann-Klinik Bad Rothenfelde, Bad Rothenfelde, Germany; Department of Cardiac Surgery, Herzzentrum Dresden GmbH Universitätsklinik an der Technischen Universität Dresden, Dresden, Germany; Department of Cardiology, Internistisches Klinikum München Süd, München, Germany; Department of Cardiac Surgery, München Klinik Bogenhausen, München, Germany; Department of Cardiovascular Surgery, Universitätsklinikum Gießen, Gießen, Germany; Department of Cardiac Surgery, Krankenhaus der Barmherzigen Brüder, Trier, Germany; Department of Cardiac Surgery, Asklepios Klinikum Harburg, Hamburg, Germany; Department of Cardiothoracic and Vascular Surgery, Deutsches Herzzentrum der Charité (DHZC), Berlin, Germany; Charité—Universitätsmedizin Berlin, corporate member of Freie Universität Berlin, Humboldt-Universität zu Berlin, and Berlin Institute of Health, Berlin, Germany; German Center for Cardiovascular Research (DZHK), Partner Site Berlin, Berlin, Germany

**Keywords:** Atrial fibrillation, Long-standing persistent, Surgical ablation, Registry

## Abstract

**OBJECTIVES:**

The CArdioSurgEry Atrial Fibrillation (CASE-AF) registry is a prospective, multicentre study for collecting and analysing real-world data of surgical atrial fibrillation (AF) treatment. This study aimed to evaluate outcomes of surgery for long-standing persistent AF at 1 year.

**METHODS:**

In total, 17 centres consecutively include all eligible patients with continuous AF lasting for ≥1 year. Exclusion criteria are missing informed consent or age <18 years. For patient-reported outcomes measures, the European Heart Rhythm Association score was used. No presence of AF (based on ECG findings including Holter ECG and/or implanted devices), no re-ablation, no further cardioversion and no rehospitalization due to AF after a 3-month blanking period defined no AF recurrence at 1 year.

**RESULTS:**

From January 2017 to January 2020, a total of 1115 patients were enrolled in CASE-AF. Of them, 202 patients (mean age 69.7 ± 7.8 years, 27.2% female) underwent surgical ablation of long-standing persistent AF (study cohort), mostly accompanied by left atrial appendage closure (*n* = 180 [89%], resection *n* = 75 [42%]) and predominantly performed as concomitant (*n* = 174 [86%]) and left atrial only procedure (*n* = 144 [71%]). Early mortality (30 days) was 2.0% and morbidity was low. At follow-up (median 14.4 months, interquartile range, 12.7–17.6 months, 100% complete), 106 patients (56%) had no AF recurrence and 93% of them were asymptomatic. AF recurrence was accompanied by AF-related rehospitalization (*n* = 12, *P* = 0.003), direct current shock cardioversion (*n* = 23, *P* < 0.001), AF ablation (*n* = 7, *P* = 0.003) and stroke (*n* = 3, *P* = 0.059). Multivariable analysis identified cryoablation, predominantly performed endocardially including additional left atrial (74%) and biatrial (42%) lesions, as a significant factor for freedom from AF recurrence (odds ratio 2.7, 95% confidence interval 1.07–6.79, *P* = 0.035).

**CONCLUSIONS:**

According to CASE-AF, surgical ablation of long-standing persistent AF is most effective when concomitantly performed using endocardial cryoablation. Ongoing follow-up allows further elucidation of efficacious treatment strategies.

**Figure:**
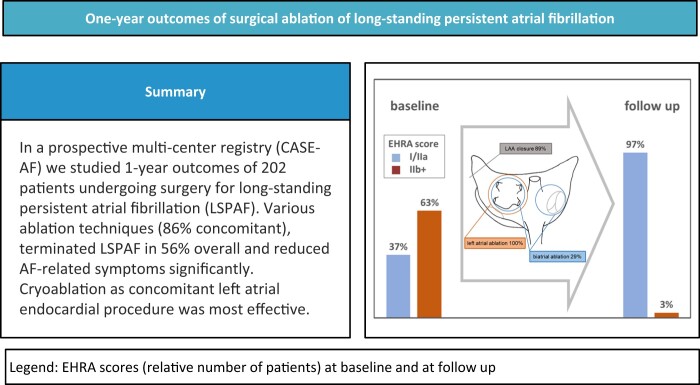

## INTRODUCTION

The prevalence of atrial fibrillation (AF), being associated with higher risk of stroke, heart failure and premature death, increases with an ageing population [1]. AF is present in a significant number of patients requiring cardiac surgery and was shown to worsen prognosis in patients undergoing surgery for valvular and coronary heart disease [2–4]. In this cohort, concomitant AF surgery results in an increased freedom from AF, atrial flutter and atrial tachycardia [5]. Long-standing persistent AF (LSPAF) is associated with electrical, functional and structural alterations of the atria. Due to its chronic nature and advanced disease stage, LSPAF is more difficult to treat and outcomes are typically worse [6–10].

After introduction of the cut-and-sew Cox maze operation for surgical AF treatment >30 years ago [11], the evolution of ablation technology and adaptions to electrophysiological findings [10, 12, 13] yielded numerous modifications, e.g. lesion patterns and atrial approaches. Today, growing experience with a variety of techniques for AF ablation and evidence have led to guideline recommendations [1, 14, 15]. Nonetheless, reports analysing procedures and results in current clinical practice are limited. The CArdioSurgEry Atrial Fibrillation (CASE-AF) registry, an ongoing nationwide (Germany), prospective, observational, multicentre study was established for collecting and analysing real-world data of surgical AF treatment [16]. The objective of this study was to evaluate techniques and 1-year outcomes of surgery for LSPAF.

## PATIENTS AND METHODS

### Ethics statement

The study was approved by the Ethics Committee on 6 May 2016 (Landesärztekammer Rheinland-Pfalz, ID number 837.536.15 [10304]). Formal written consent was obtained from all included patients.

### CArdioSurgEry Atrial Fibrillation registry

The CASE-AF registry (ClinicalTrials.gov identifier: NCT03091452, registered March 27, 2017) is a multicentre observational study, which prospectively collects longitudinal data of patients (age >18 years) undergoing concomitant or stand-alone AF surgery in Germany (inclusion criteria) [16]. In total, 17 cardiothoracic centres consecutively include all eligible patients. The criteria for surgical AF treatment depended on case-by-case decisions in the respective centres. Exclusion criteria were missing informed consent or age <18 years. The first follow-up was performed at the study sites after 12 months. Collected data included arrhythmia documentation by ECG as well as by Holter ECG and/or by cardiac implanted electronic devices (*n* = 85). Preoperative, procedural, postoperative and follow-up data are entered in web-based electronic case report forms. Data management and analysis is performed by the Institut für Herzinfarktforschung (IHF, Ludwigshafen, Germany).

### Patients

**Figure 1:**
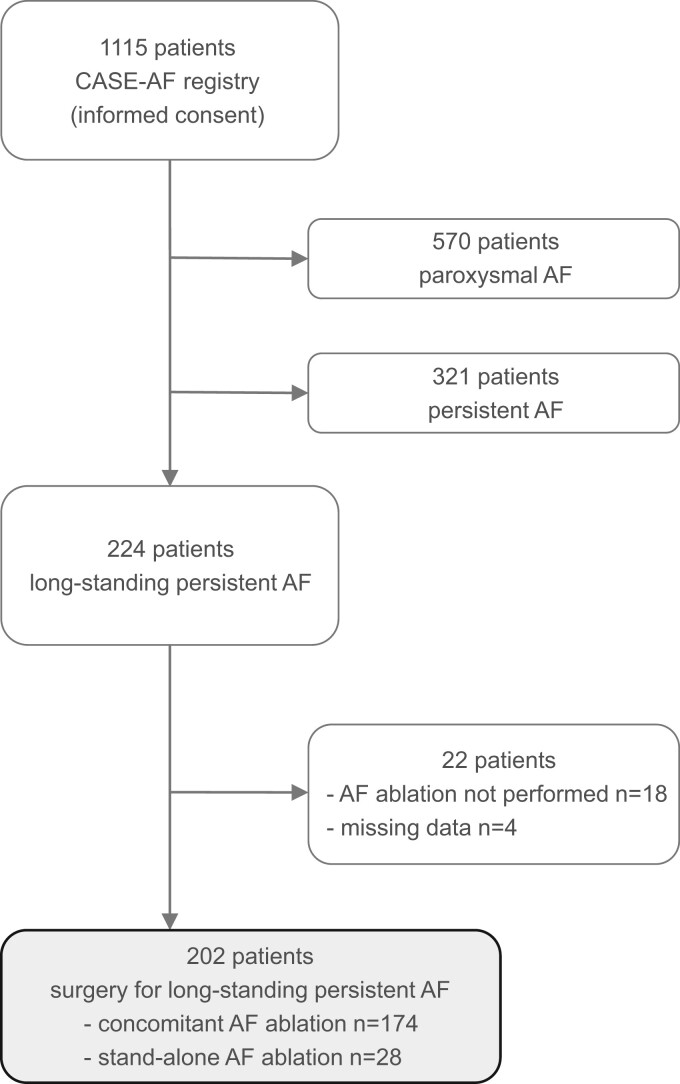
Flow diagram of the CASE-AF registry population. According to STROBE guidelines [17], the flow diagram reports the number of patients included in the CASE-AF registry as well as the number and reasons (details see text) of exclusions explaining how the final study cohort of 202 patients undergoing surgical ablation of long-standing persistent AF was arrived at. AF, atrial fibrillation; CASE-AF: CArdioSurgEry Atrial Fibrillation.

From January 2017 to January 2020, 1115 patients were enrolled in the CASE-AF registry. Figure 1 depicts the flow diagram of the registry population according to strengthening the reporting of observational studies in epidemiology (STROBE) guidelines [17]. The final cohort of this study consisted of 202 patients with LSPAF undergoing concomitant or stand-alone surgical AF ablation. No patient underwent a classical Cox-maze procedure. Reasons why AF ablation was not commenced (*n* = 17) or discontinued (*n* = 1) were advanced atrial dilatation (*n* = 6, 33%), adhesions and/or unfavourable anatomy (*n* = 5, 28%), surgeon’s discretion (*n* = 1, 6%) and others (*n* = 6, 33%).

### Definitions

AF was classified as long-standing persistent, if continuous AF lasts for ≥1 year [1, 16]. The incidences (≤30 days) of death, myocardial infarction and stroke were combined as major adverse cardiac and cerebrovascular event rate and defined operative outcomes. Success of surgical LSPAF ablation at 1 year was defined as no AF recurrence summarizing no presence of AF lasting longer than 30 s based on ECG findings (including Holter ECG and/or cardiac implanted electronic devices), no re-ablation, no further cardioversion and no rehospitalization due to AF after an initial blanking period of 3 months [14]. For patient-reported outcomes measures (PROMs), the European Heart Rhythm Association (EHRA) score was used at baseline and at 12-month follow-up [18].

### Data analysis

Data reporting and statistical analysis followed published definitions and guidelines [17, 19]. If missing data were present, summary statistics are case-complete statistics. Categorical variables are presented as absolute and relative frequencies. For continuous data, means and standard deviations or if there was evidence of the distribution of data being non-normal medians with lower and upper quartiles were calculated, respectively. For comparisons between patients with/without AF recurrence, continuous variables were analysed using the Mann–Whitney–Wilcoxon test and categorical data by Pearson Chi-squared test. For identification of relevant factors associated with successful rhythm outcome (no AF recurrence) multivariable analysis was performed by binary logistic regression using predefined demographic, clinical and procedural covariates (see Fig. 4). A *P*-value of <0.05 was considered to be statistically significant. All the statistical analyses were performed by a biostatistician (Taoufik Ouarrak) using SAS software (Version 9.4, SAS Institute Inc., Cary, NC, USA).

## RESULTS

### Preoperative status

Table 1 lists the baseline characteristics of the study cohort. According to EHRA score, arrhythmia-related symptoms were frequently present. A large number of patients presented with heart valve disease and heart failure symptoms. LSPAF was accompanied by left atrial dilatation as well as an increased risk of thromboembolic Congestive heart failure, Hypertension, Age, Diabetes mellitus, Stroke, Vascular disease, Sex category (CHA_2_DS_2_-VASc score) and bleeding events Hypertension, Abnormal renal and liver function, Stroke, Bleeding, Labile INR, Elderly, Drugs (HAS-BLED score). A significant number of patients had undergone previous AF treatment [direct current (DC) shock cardioversion and/or catheter ablation] and/or presented as non-responders to antiarrhythmic treatment with amiodarone.

**Table 1:** Baseline characteristics

	Surgical LSPAF ablation, *n* = 202
Age (years), mean (SD)	69.7 (7.8)
Female, % (*n*/*n*)	27.2 (55/202)
Body mass index (kg/m^2^), mean (SD)	29.4 (20.0), *n* = 201
Leading valvular heart disease, % (*n*/*n*)	63.5 (125/197)
LVEF ≤40%, % (*n*/*n*)	18.3 (37/202)
LA diameter >45 mm, % (*n*/*n*)	69.3 (113/163)
NYHA class ≥III, % (*n*/*n*)	58.1 (108/186)
EHRA score >IIb, % (*n*/*n*)	61.6 (101/164)
CHA_2_DS_2_-VASc score ≥2, % (*n*/*n*)	88.9 (169/190)
HAS-BLED score ≥3, % (*n*/*n*)	36.8 (70/190)
Resistance to amiodarone, % (*n*/*n*)	28.3 (35/147)
DC shock cardioversion (≥1), % (*n*/*n*)	45.3 (91/201)
Catheter ablation (≥1), % (*n*/*n*)	20.9 (41/196)
Pacemaker/ICD, % (*n*/*n*)	7.9 (16/202)
Myocardial infarction, % (*n*/*n*)	10.9 (22/201)
Stroke, % (*n*/*n*)	6.0 (12/201)
TIA, % (*n*/*n*)	6.0 (12/201)
Peripheral arterial disease, % (*n*/*n*)	5.5 (11/201)
Renal failure, % (*n*/*n*)	26.9 (54/201)
Chronic pulmonary disease, % (*n*/*n*)	7.5 (15/201)
Liver disease, % (*n*/*n*)	4.0 (8/201)
Arterial hypertension, % (*n*/*n*)	82.6 (166/201)
Diabetes, % (*n*/*n*)	19.4 (39/201)
EuroSCORE II, mean (SD)	5.4 (9.9), *n* = 200

DC: direct current; EHRA: European Heart Rhythm Association; EHRA score: modified European Heart Rhythm Association symptom scale [18]; ICD: implanted cardioverter defibrillator; LA: left atrial; LSPAF: long-standing persistent atrial fibrillation; LVEF: left ventricular ejection fraction; NYHA: New York Heart Association; SD: standard deviation; TIA: transient ischaemic attack; CHA_2_DS_2_: Congestive heart failure, Hypertension, Age, Diabetes mellitus, Stroke, Vascular disease, Sex category; HAS-BLED: Hypertension, Abnormal renal and liver function, Stroke, Bleeding, Labile INR, Elderly, Drugs.

### Procedures

**Figure 2:**
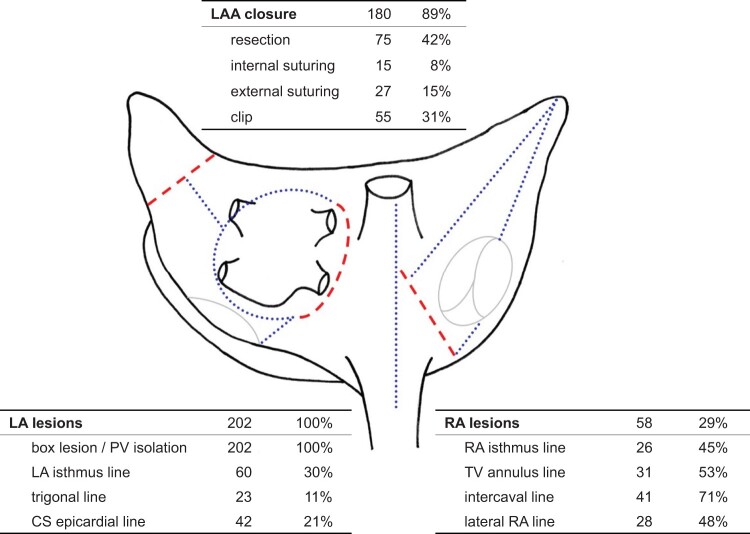
Atrial procedures performed for surgical ablation of LSPAF. The picture shows the posterior view of the left and right atrium with the schematic Cox Maze lesion set (red interrupted lines: surgical incisions, blue dotted lines: ablation lines). Tables list actual atrial procedures (*n*, %) performed in the study cohort for surgical LSPAF treatment. CS: coronary sinus; LA: left atrial; LAA: left atrial appendage; PV: pulmonary vein; RA, right atrial; TV: tricuspid valve.

In 174 patients (86%), surgical LSPAF ablation was a concomitant and in 28 patients (14%) a stand-alone procedure. There were 4 re-operations (2.0%). Operative data are summarized in Table 2 and Fig. 2. In 73%, the surgical approach was a median sternotomy, whereas minimally invasive thoracotomy or thoracoscopy was used in 27%, essentially in stand-alone and mitral valve (MV) procedures. Cardiopulmonary bypass and cardioplegic arrest were used during ablation in 170 (84%) and 120 cases (61%), respectively. In concomitant procedures, the leading indications for surgery were heart valve diseases, with 50% most frequently involving the MV. Coronary artery bypass graft surgery was performed in 30% of patients. The energy source used for tissue ablation was cryothermia in 59% (94% endocardial ablation) and radiofrequency energy in 41% (100% epicardial, 94% bipolar ablation). Whereas ablation targeted the left atrium in all patients, a biatrial lesion set was applied in 29% overall (Fig. 2). Left atrial only ablation was most frequently performed in MV surgery (± coronary artery bypass graft surgery) and in stand-alone procedures (Table 2). With cryoablation, a biatrial procedure was performed in 42% of patients. As shown in Fig. 2, the left atrial appendage (LAA) was surgically treated in 89% of patients. The LAA was resected in 42% overall, most frequently in association with combined aortic valve and MV surgery (Table 2).

**Table 2:** Procedural details of surgical long-standing persistent atrial fibrillation ablation

	MV surgery (± CABG), *n* = 38	AV surgery (± CABG), *n* = 48	AV + MV surgery (± CABG), *n* = 14	MV + TV surgery (± CABG), n = 35	CABG, *n* = 32	Other concomitant procedures, *n* = 7	Stand-alone procedures, *n* = 28
Median sternotomy, % (*n*/*n*)	53 (20/38)	98 (47/48)	100 (14/14)	69 (24/35)	100 (32/32)	100 (7/7)	14 (4/28)
MIS—lateral thoracotomy, % (*n*/*n*)	45 (17/38)	2 (1/48)	–	29 (10/35)	–	–	4 (1/28)
MIS—thoracoscopy, % (*n*/*n*)	3 (1/38)	–	–	3 (1/35)	–	–	82 (23/28)
CPB for ablation	97 (37/38)	100 (48/48)	93 (13/14)	100 (35/35)	81 (26/32)	100 (7/7)	14 (4/28)
Cardioplegic arrest for ablation , % (*n*/*n*)	82 (31/38)	58 (26/45)	79 (11/14)	88 (30/34)	39 (12/31)	86 (6/7)	14 (4/28)
Cryoablation, % (*n*/*n*)	90 (34/38)	48 (23/48)	86 (12/14)	97 (34/35)	19 (6/32)	86 (6/7)	14 (4/28)
Radiofrequency ablation , % (*n*/*n*)	11 (4/38)	54 (26/48)	14 (2/14)	3 (1/35)	81 (26/32)	29 (2/7)	86 (24/28)
Biatrial ablation, % (*n*/*n*)	13 (5/38)	23 (11/48)	21 (3/14)	66 (23/35)	22 (7/32)	71 (5/7)	14 (4/28)
Left atrial only ablation, % (*n*/*n*)	87 (33/38)	77 (37/48)	79 (11/14)	34 (12/35)	78 (25/32)	29 (2/7)	86 (24/28)
LAA closure, % (*n*/*n*)	84 (32/38)	98 (47/48)	93 (13/14)	83 (29/35)	94 (29/31)	100 (7/7)	82 (23/28)
LAA resection, % (*n*/*n*)	32 (12/38)	50 (24/48)	71 (10/14)	23 (8/35)	52 (16/31)	57 (4/7)	4 (1/28)

AV: aortic valve; CABG: coronary artery bypass grafting; CPB: cardiopulmonary bypass; LA: left atrial; LAA: left atrial appendage; MIS: minimally invasive surgery; MV: mitral valve; TV: tricuspid valve.

### Early outcomes

There was no death or injury on the oesophagus, pulmonary veins or circumflex artery in association with the ablation procedure. In 1 patient (0.6%) undergoing concomitant LSPAF ablation, accidental injury of the inferior vena cava occurred and could be managed surgically without persistent damage. Events defining perioperative mortality and morbidity are listed in Table 3. No major adverse cardiac and cerebrovascular event occurred after stand-alone LSPAF ablation. No patient presented with postoperative cardiac failure. The leading indications for implantation of a new pacemaker/implanted cardioverter defibrillator were complete atrioventricular block (*n* = 11) and sinus arrest (*n* = 4). At discharge, sinus rhythm was present in 61% overall.

**Table 3:** Perioperative data (≤30 days)

	Surgical LSPAF ablation, *n* = 202
MACCE, % (*n*/*n*)	4.5 (9/200)
Death	2.0 (4/200)
Myocardial infarction	1.0 (2/201)
Stroke	2.0 (4/201)
TIA, % (*n*/*n*)	0.5 (1/201)
Severe bleeding complication, % (*n*/*n*)	2.0 (4/201)
Re-exploration, % (*n*/*n*)	5.5 (11/201)
Pericardial effusion, % (*n*/*n*)	4.5 (9/201)
Renal failure, % (*n*/*n*)	3.0 (6/201)
Respiratory failure/pneumonia, % (*n*/*n*)	2.5 (5/201)
Sternal wound infection, % (*n*/*n*)	1.5 (3/201)
New pacemaker/ICD, % (*n*/*n*)	10.4 (21/201)
single chamber pacemaker	1.0 (2/201)
dual chamber pacemaker	8.0 (16/201)
ICD	1.0 (2/201)
CRT	0.5 (1/201)
DC shock cardioversion, % (*n*/*n*)	12.4 (25/201)
Class-III antiarrhythmic drugs, % (*n*/*n*)	29.1 (58/199)
Sinus rhythm at discharge, % (*n*/*n*)	60.5 (118/195)
Postoperative hospital stay, median (LQ, UQ)	10 (8, 15)

CRT: cardiac resynchronization therapy; DC: direct current; ICD: implanted cardioverter defibrillator; LQ: lower quartile; LSPAF: long-standing persistent atrial fibrillation; MACCE: major adverse cardiac and cerebrovascular events; TIA: transient ischaemic attack; UQ: upper quartile.

### Late outcomes

**Figure 3:**
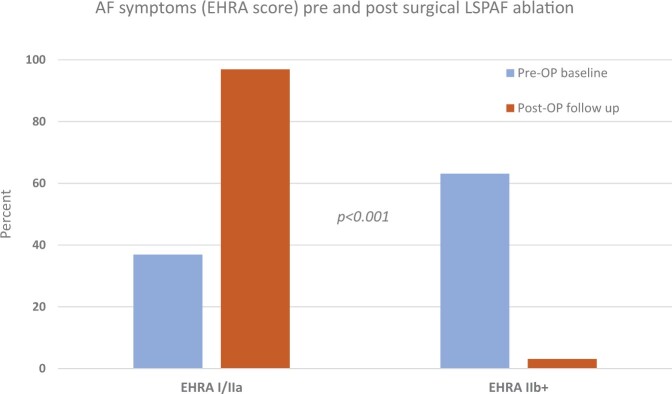
Patient-reported outcome regarding AF-related symptoms (EHRA score) [18]. Moderate, severe or disabling AF-related symptoms (EHRA score ≥IIb) have been reported by 82 patients (63%) prior to surgical LSPAF ablation compared to 4 patients (3%) at follow-up (*P* < 0.001). Only 130 patients with complete EHRA score data have been included in this analysis. EHRA score I: no symptoms; EHRA score IIa/b: mild/moderate symptoms, normal daily activity not affected; EHRA score III: severe symptoms, normal daily activity affected; EHRA score IV: disabling symptoms, normal daily activity discontinued. AF: atrial fibrillation; EHRA: European Heart Rhythm Association; LSPAF: long-standing persistent atrial fibrillation.

**Figure 4:**
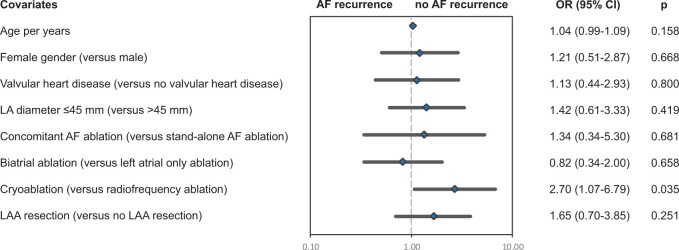
Multivariable analysis for heart rhythm outcomes at 1-year follow-up. The results of binary logistic regression analysis of predefined demographic, clinical and procedural covariates are shown. The graphs depict the odds ratios (rhombs) with their lower and upper confidence limits on a logarithmic axis. The interrupted vertical line indicates an odds ratio of 1 (‘no effect’). The quality of the multivariable model was assessed by ROC curve (AUC 0.70). AF: atrial fibrillation; AUC: area under the curve; CI: confidence interval; LA: left atrial; LAA: left atrial appendage; OR: odds ratio; ROC: receiver operating characteristic.

The median follow-up was 14.4 months (interquartile range, 12.7–17.6 months). Patient-reported outcome regarding AF-related symptoms (EHRA score) showed significant improvement (Fig. 3) and 106 patients (56%) were free of AF according to the definition used. Late results regarding rhythm outcomes are summarized in Table 4. Death, stroke and TIA did not occur after stand-alone procedure. AF recurrence was associated with a significantly increased rehospitalization rate, in particular due to AF (*n* = 12, *P* = 0.003) and repeat AF treatment (*n* = 23 DC shock cardioversion, *P* < 0.001, *n* = 7 AF ablation, *P* = 0.003). Moreover, there was a statistical trend to an increased stroke rate (Table 4). Patients in whom AF did not recur reported none (EHRA score I) or only mild AF-related symptoms (not affecting normal daily activity, EHRA score IIa), in 69 (93%) and 5 (7%) cases, respectively. In comparison, patients with AF recurrence presented with EHRA score I, IIa, IIb and III in 17 (22%), 53 (70%), 4 (5%) and 2 (3%) cases (*P* < 0.001). Regarding factors being associated with successful rhythm outcome (no AF recurrence), Fig. 4 depicts the results of multivariable testing. Apart from cryoablation, none of the assessed demographic, clinical and procedural covariates exhibited a relevant association with rhythm outcomes at 12 months. Not more than a weak and non-significant signal was seen for LAA excision predicting the absence of AF recurrence.

**Table 4:** Outcomes at 1 year

	All patients, *n* = 191	No AF during follow-up,^a^*n* = 106	AF recurrence, *n* = 85	*P*-value
Mortality,^b^ % (*n*/*n*)	1.6 (3/191)	2.8 (3/106)	0 (0/85)	0.118
Stroke,^b^ % (*n*/*n*)	1.6 (3/184)	0 (0/99)	3.5 (3/85)	0.059
TIA,^b^ % (*n*/*n*)	1.1 (2/183)	2.0 (2/99)	0 (0/84)	0.190
Severe bleeding complication,^b^ % (*n*/*n*)	2.7 (5/182)	1.0 (1/97)	4.7 (4/85)	0.130
New pacemaker/ICD,^b^ % (*n*/*n*)	11.5 (21/183)	8.1 (8/99)	15.5 (13/84)	0.118
VVI	1.1 (2/183)	0 (0/99)	2.4 (2/84)	0.123
DDD	4.4 (8/183)	5.1 (5/99)	3.6 (3/84)	0.626
ICD	4.9 (9/183)	3.0 (3/99)	7.1 (6/84)	0.200
CRT	1.1 (2/183)	0 (0/99)	2.4 (2/84)	0.123
Rehospitalization, % (*n*/*n*)	43.5 (80/184)	31.3 (31/99)	57.6 (49/85)	<0.001
EHRA score >IIb, % (*n*/*n*)	4.0 (6/150)	0 (0/74)	7.9 (6/76)	<0.001
Class-III antiarrhythmic drugs, % (*n*/*n*)	9.8 (18/184)	11.1 (11/99)	8.2 (7/85)	0.513
Betablocker, % (*n*/*n*)	81.5 (150/184)	83.8 (83/99)	78.8 (67/85)	0.382
Anticoagulation, % (*n*/*n*)	70.7 (130/184)	62.6 (62/99)	80.0 (68/85)	0.010

a No AF recurrence was defined as no presence of AF, no re-ablation, no further cardioversion and no rehospitalization due to AF after an initial blanking period of 3 months.

b Post-hospital incidence rate.

AF: atrial fibrillation; CRT: cardiac resynchronization therapy; EHRA: European Heart Rhythm Association; ICD, implanted cardioverter defibrillator; TIA: transient ischaemic attack.

## DISCUSSION

Out of 1115 CASE-AF registry patients, 18% underwent surgical ablation of LSPAF. Mostly, these were concomitant procedures in association with heart valve surgery. Operative mortality and morbidity were low. At 1 year, AF was not present and did not recur in the meantime (no repeat cardioversion or ablation, no rehospitalization due to AF) in 56% of patients and 93% of these were asymptomatic. In comparison, a recent meta-analysis of 113 studies (18 657 patients, mean age 59 ± 3 years) demonstrated a 43% chance of maintaining sinus rhythms off antiarrhythmic drugs with catheter ablation of persistent (52 ± 22%) and LSPAF at 25 ± 12 months follow-up [20]. Regarding AF treatment in general, its success is determined by AF duration and the degree of atrial remodelling [6–10]. Very recently, the analysis of all CASE-AF patients confirmed that persistent AF *per se* is a strong predictor for AF recurrence [21]. Hence, termination of LSPAF is most difficult and the results of this study are encouraging, in particular as almost all successfully treated patients reported no AF-related symptoms at follow-up (Fig. 3). Noteworthy, AF recurrence herein was not only accompanied by arrhythmia-related symptoms, rehospitalization and repeat DC shock cardioversion and/or catheter ablation but also indicated an increased stroke rate.

Multivariable analysis identified cryoablation as an important predictor for successful LSPAF ablation (Fig. 4). This favourable outcome for cryoablation over radiofrequency ablation can be explained by the fact that cryoablation was performed endocardially in 94% and, apart from PV isolation, included additional left atrial and biatrial lesions in 74% and 42%. Within the scientific community, there is a persisting debate whether LSPAF ablation should target both atria, the left atrium, or only the pulmonary veins [8, 20, 22]. Based on the idea that macro-reentry circuits and structural atrial remodelling as the dominating pathophysiological mechanisms sustaining this type of arrhythmia affect both atria, it is conclusive that biatrial lesion patterns or the full biatrial Cox maze IV lesion set may be more effective in persistent and LSPAF [8, 22]. Others argue that the left atrium is usually the electrical driving chamber [10, 12]. Thus, data demonstrate that LSPAF is frequently successfully treated by lesion sets applied to the left atrium only [6]. Also with this study, there is evidence that the addition of atrial lesions to PV isolation as reflected by performing cryoablation was important, but biatrial surgical ablation could not be demonstrated to be more effective regarding LSPAF treatment (Fig. 4). This is in line with findings from Soni *et al.*, who showed that right atrial lesions do not improve the efficacy of a complete left atrial lesion set in the surgical treatment of AF [23]. Furthermore, these authors found an increased procedural morbidity when right atrial lesions were added [23]. When balancing the extent of surgical ablation with the complexity and risks of the whole procedure, one has to consider the effect of correcting an underlying heart disease, the primary indication for surgery in concomitant procedures, on rhythm outcome as well. This is one of the reasons why the AF recurrence rate was lower after concomitant (39%) than after stand-alone LSPAF ablation (75%) in this study. Undoubtedly, a surgical stand-alone procedure is thought to be a part of a multi-step or single hybrid approach, thus being able to treat LSPAF successfully in 60–80% of patients [24, 25].

Considering that the present registry data suggest an association between the leading surgical procedure and the ablation technique used, the need for further research on surgical LSPAF treatment in different indications for primary surgery emerges. Together with continuous education and training for surgeons performing AF ablation, this will lead to more standardized procedures and steady results.

Although LAA surgery was performed in almost all patients, higher rates (>50%) of LAA resection were only observed in concomitant procedures using a median sternotomy (Table 2). Regarding the indicated association between LAA excision and the absence of AF recurrence (Fig. 4), it is important to consider, that LAA resection is part of the Cox maze procedure aiming to reduce the left atrial mass and to obliterate the substrate of reentry circles [11]. Apart from arrhythmia treatment, LAA resection/occlusion is important for the prevention of thromboembolic events during episodes of AF, in particular stroke. In this study, however, only one of the three patients with AF recurrence and stroke (Table 4) had no LAA surgery, but it is definite that such low incidence prohibits any conclusion. Regarding stroke prevention, future studies must elucidate, if occlusion, e.g. external clipping, is as effective as LAA resection.

### Limitations

Potential selection bias and unmeasured or uncontrolled confounders are the most important limitations inherent to observational studies. For instance, the CASE-AF registry does not contain information about patients with LSPAF who underwent cardiac surgery, but not AF ablation or about the relationship between rhythm outcome and results of heart valve surgery. Thus, the results of this study cannot be generalized to all patients with LSPAF. Considering that (i) restrictions analysing more characteristics statistically resulted from the limited cohort size and (ii) only factors (associated to the outcome) can be detected amongst the covariates that have been collected heretofore imply that statistical analysis can only reduce but not eliminate biases. As the exact time points of the first AF recurrence (within the limited interval of follow-up) were too imprecisely or missing in about 50% of cases, time-to-event analyses have not been used.

Nonetheless, registry data are considered the gold standard of observational data and second best to randomized clinical trial data. In contrast to randomized clinical trials, registries provide important information that reflect common practice and the general target population. Thus, CASE-AF is a considerable large registry gathering procedural and follow-up data of surgical AF ablation. Although the inhomogeneity of the cohort and the current time of follow-up limit statements regarding long-term outcomes, CASE-AF is able to overcome this limitation in future, as the registry is ongoing. Furthermore, reporting incidence rates will become more realistic with more patients and longer follow-up. Despite developments in statistical methodology, inferring causal effects with registry data remains difficult.

## CONCLUSIONS

CASE-AF provides valuable information on current surgical ablation of LSPAF. Using various techniques, it is predominantly performed as concomitant procedure comprising LAA surgery. Overall, 56% of patients show no AF recurrence at 1 year and the vast majority of them is free of AF-related symptoms. Only for cryoablation, usually performed as left atrial endocardial procedure, a significant association with favourable rhythm outcome was demonstrated. With its real-world perspective and ongoing follow-up, CASE-AF allows further elucidation of efficacious strategies for surgical LSPAF ablation.

## Data Availability

The data underlying this article cannot be shared publicly due to data protection regulations. The data can be shared on reasonable request to the corresponding author.

## ABBREVIATIONS

AF: Atrial fibrillation
CASE-AF: CArdioSurgEry Atrial Fibrillation registry
DC: Direct current
EHRA: European Heart Rhythm Association
LAA: Left atrial appendage
LSPAF: Long-standing persistent atrial fibrillation
